# Reference values for white blood-cell-based inflammatory markers in the Rotterdam Study: a population-based prospective cohort study

**DOI:** 10.1038/s41598-018-28646-w

**Published:** 2018-07-12

**Authors:** Jesse Fest, Rikje Ruiter, M. Arfan Ikram, Trudy Voortman, Casper H. J. van Eijck, Bruno H. Stricker

**Affiliations:** 1 Department of Surgery, Erasmus MC University Medical Center, Rotterdam, The Netherlands; 2 Department of Epidemiology, Erasmus MC University Medical Center, Rotterdam, The Netherlands

## Abstract

Novel prognostic inflammatory markers of cancer survival and cardiovascular disease are; the neutrophil-to-lymphocyte ratio (NLR), the platelet-to-lymphocyte ratio (PLR) and the systemic immune-inflammation index (SII). As normal values for these markers are unknown, our objective was to obtain reference values in the general population. We obtained data from a population-based prospective cohort study of individuals aged 45 years and over between 2002 and 2014. Absolute blood counts were used to calculate the NLR, PLR and SII. All inflammatory indices followed a log-normal distribution. We calculated the mean and 95% reference intervals in an unselected population. Furthermore we studied whether the inflammatory markers differed between age categories and gender. In total 8,711 participants (57.1% female; mean age 65.9 years, standard deviation 10.5 years) were included. Mean values and corresponding 95% reference intervals for the NLR were: 1.76 (0.83–3.92), for PLR: 120 (61–239) and for SII: 459 (189–1168). The inflammatory markers increased with age. The PLR and SII were higher in females, whilst the NLR was higher in males. In conclusion, we provided reference values for new inflammatory markers. All increase with age and vary with gender. This provides context that allows for proper interpretation of their potential value in future clinical practice and research.

## Introduction

Low-grade inflammation is associated with important chronic diseases in the elderly such as diabetes, cardiovascular disease and cancer^1–7^. For instance, several immune mechanisms play a role in the formation and activation of atherosclerotic plaques that lead up to cardiovascular disease and the over-expression of TNF-α is associated with insulin resistance and subsequently type 2 diabetes^2,7^. Furthermore chronic inflammation is also since long considered as one of the basic pathogenic processes in cancer development^3,4^. Additionally, it is thought that, once the cancer has developed, the immune system plays an important role in surveillance and elimination of cancer cells^4^.

This has led to the examination of various inflammatory markers and indices as a potential biomarker or prognostic factors^8^. Traditional measures, such as C-reactive protein (CRP) and the erythrocyte sedimentation rate (ESR) have been extensively studied, previously^5,6,8^. Recently, several new white blood-cell-based inflammatory indices have been introduced as prognostic markers: the neutrophil-to-lymphocyte ratio (NLR), the platelet-to-lymphocyte ratio (PLR) and the systemic immune inflammation index (SII)^9–13^.

Both the NLR and PLR are ratios; of the peripheral neutrophil and lymphocyte counts and the peripheral platelet and lymphocyte counts, respectively. The SII has integrated peripheral lymphocyte, neutrophil and platelet counts into one indicator, with the aim to better reflect the balance between the host’s inflammatory and immune status^10^. The NLR, PLR and SII can be easily calculated from low-cost and frequently used available measures and are thought to be more specific than CRP or the ESR.

It is generally assumed that the levels of these inflammatory markers are elevated in individuals with cardiovascular disease or cancer. However, normal ranges for the NLR, PLR or SII are unknown and most researchers have estimated cut-off points within their sample population, resulting in a wide and inconsistent range of cut-off points used in current literature^12–14^. Reference values are therefore needed to put the results of previous studies into a context that allows for proper interpretation of their potential clinical value. The objective of this study was therefore, to obtain these reference values from the general population in a large and longstanding population-based prospective cohort study.

## Methods

### Study setting

The analyses were performed in the Rotterdam Study, a long term population based prospective cohort study in the Rotterdam area, the Netherlands. Its rationale and design have been described extensively, previously^15,16^. Briefly, inhabitants of the suburb Ommoord, aged 55 years and older, were invited to participate in 1989. Of the 10,275 invited subjects, 7,983 entered the study (78%). A second cohort of 3,011 persons (67% response), was enrolled between 2000 and 2001. In 2006 a third cohort, with 3,932 persons of 45 years and older, was enrolled (65% response). This resulted in an overall study population of 14,926 individuals, aged 45 years and older.

Participants were visited at home at baseline for a standardized interview on health status. Subsequently, a physical examination followed during a visit at the study centre. These interviews and visits were repeated approximately every four years (Supplementary Figure 1^15^). The Rotterdam Study has been approved by the institutional review board (Medical Ethics Committee) of the Erasmus Medical Center and by the review board of The Netherlands Ministry of Health, Welfare and Sports. Informed consent was obtained from all participants. All methods were performed in accordance with the relevant guidelines and regulations.

### Definition of study population

**Figure 1:**
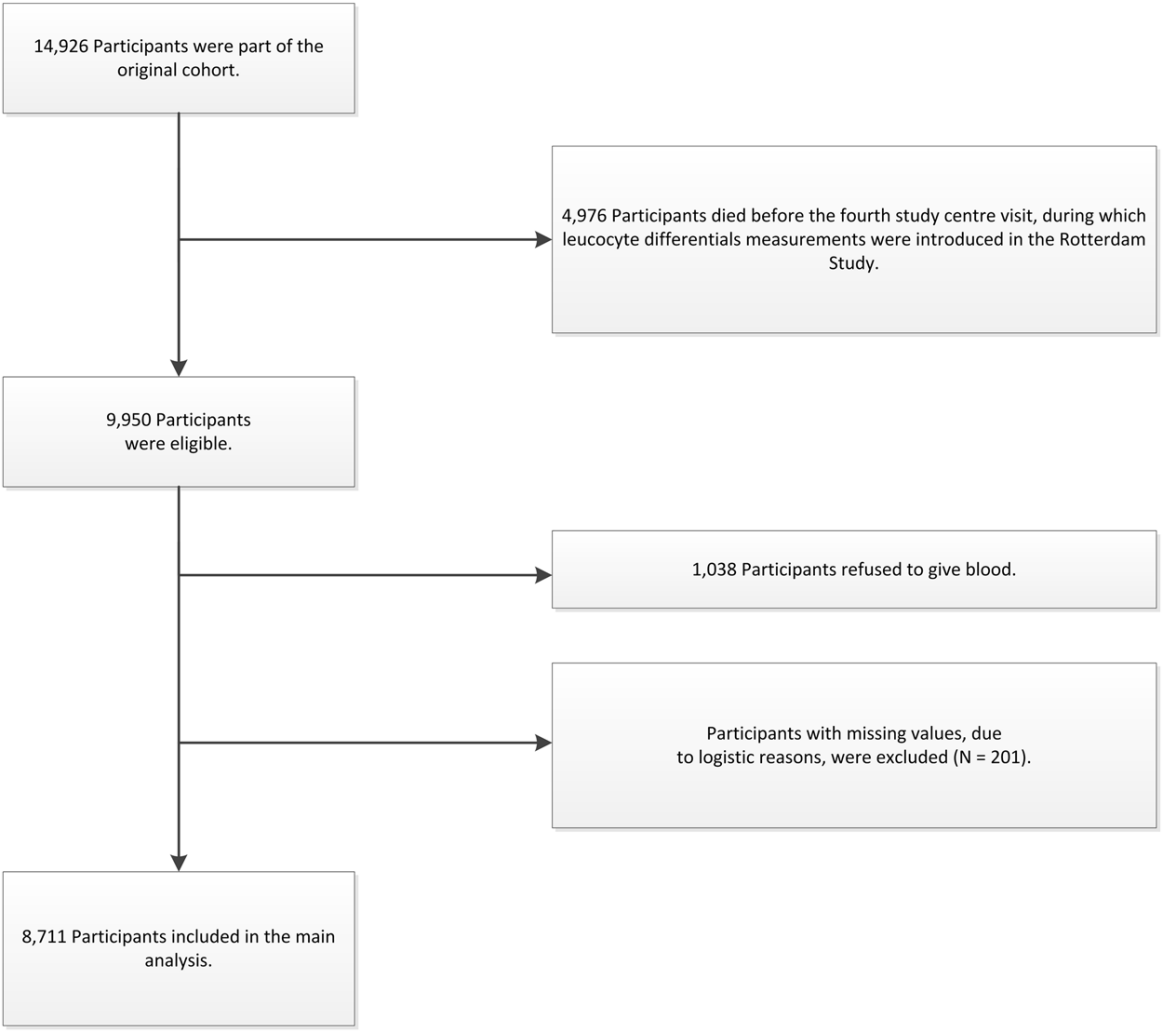
Flowchart of the study population.

White blood cell count, including leucocyte differentials, were only part of the protocol from the fourth visit of the first cohort onwards (Supplementary Figure 1^15^). Therefore, for this study we used information from the fourth centre visit of the first cohort (RS-I-4 (January 2002–July 2004); n = 3,550), the second visit of the second cohort (RS-II-2 (July 2004–December 2005); n = 2,468) and the baseline visit of the third cohort (RS-III-1 (February 2006–December 2008); n = 3,932) and onwards. Of the 9,950 eligible participants; 8,912 (89.6%) donated blood. Participants for whom the NLR, PLR or SII could not be calculated, due to missing values (n = 201), were excluded. This resulted in a study cohort of 8,711 individuals (Fig. 1).

### Collection of the samples

Fasting blood samples were collected at the study centre and were stored at −80 °C until full blood count measurements. These measurements included absolute counts of granulocytes, lymphocytes and platelets and were performed using the COULTER® Ac·T diff2™ Hematology Analyzer (Beckman Coulter, San Diego, California, USA). In an additional analysis, the normal distribution of hemoglobin and CRP levels were assessed as well. CRP levels were measured using a particle enhanced immunoturbidimetric assay (Roche Diagnostics, Mannheim, Germany).

The neutrophil-to-lymphocyte ratio was calculated on the basis of absolute peripheral granulocyte (as a proxy for the neutrophil count) (N; ×10^9^/Liter) and lymphocyte (L; ×10^9^/Liter) blood counts, using the formula: NLR = N/L^9^.

The platelet-to-lymphocyte ratio was calculated on the basis of peripheral platelet(P; ×10^9^/Liter) and lymphocyte (L; ×10^9^/Liter) blood counts, using the formula: PLR = P/L^12^.

The systemic immune-inflammation index (SII) was calculated on the basis of peripheral platelet (P; ×10^9^/Liter), granulocyte (N; ×10^9^/Liter) and lymphocyte (L; ×10^9^/Liter) blood counts, using the following formula: SII = P * N/L^10^. All the inflammatory markers are either ratios or indices and as such do not have a unit.

### Assessment of other variables

The following individual characteristics were determined at study entry interview or during the visits at the study centre: age, sex, study entry body mass index (BMI; kg/m²), smoking status (never/former/current), and socio-economic status, based on education level (SES; high [university/higher vocational education]/intermediate [general secondary education/intermediate vocational education]/low [lower secondary education/primary education with a higher, but not completed education/primary education]). Status on type 2 diabetes was ascertained either at study entry or during follow-up by use of general practitioners’ records (including laboratory glucose measurements), hospital discharge letters, and serum glucose measurements from the centre visits^17^. Diabetes was defined, in concordance with the WHO guidelines, as a fasting glucose ≥7.0 mmol/Liter or use of glucose – lowering medication^18^.

### Statistical Analyses

**Figure 2:**
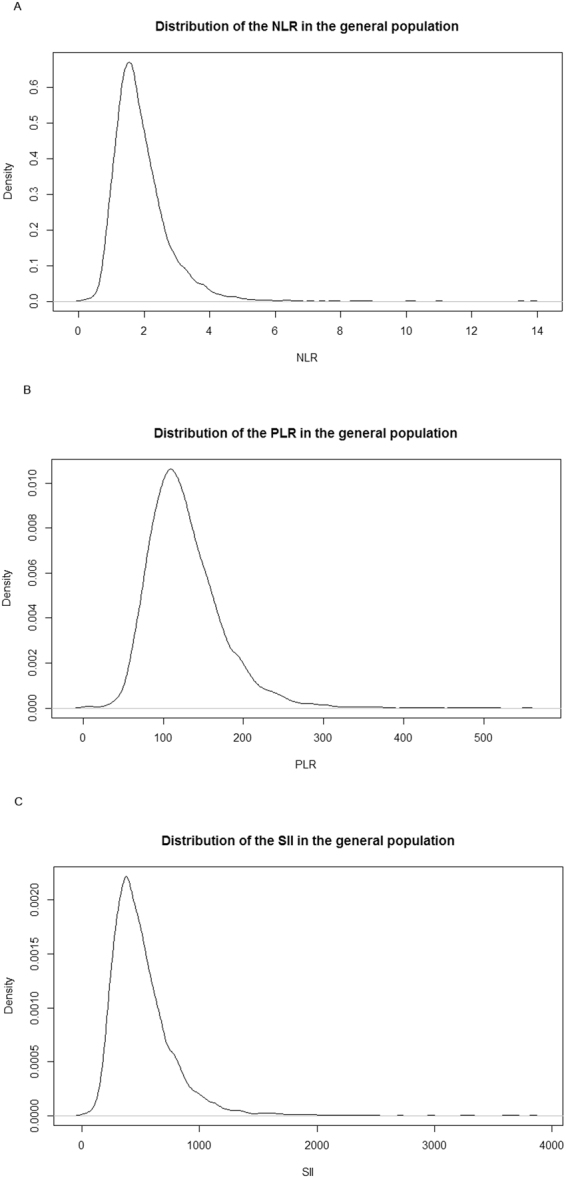
Distributions of the inflammatory markers in the general population. Panel A. NLR Panel B. PLR Panel C. SII.

The distribution of the data was visualized by means of histograms and Q-Q plots. Since none of the inflammatory markers were normally distributed and all were slightly skewed to the right (Fig. 2), we log-transformed them prior to performing any of the analyses. These values were then back-transformed to provide reference values for clinical practice^19^. To present reference values of the inflammatory markers we calculated the 2.5% and 97.5% reference limits in our study population. The 2.5% and 97.5% reference limits reflect the 2.5^th^ and 97.5^th^ percentiles, respectively. Subsequently, the differences between the distribution of the inflammatory markers in females versus males and different age classes [45–54; 55–64; 65–74; 75–84; ≥85 years], were assessed using the Student’s t-test or ANOVA.

To evaluate whether inflammatory markers indeed truly change with age we used a second measurement in the same individual, which was on average 6.1 years later (range 3.0–10.9 years), from the blood draw at RS-I-5 (March 2009–January 2011); n = 2,147; RS-II-3 (February 2011–February 2012); n = 1,893 and RS-IIII-2 (March 2012–June 2014); n = 3,122, respectively (see Supplementary Figure 1). Out of the 7,162 living participants, in total 5,849 participants had two measurements available. Differences were assessed using a Paired Samples t-test.

To see whether the distribution was influenced by any current infection, we further assessed the associations in individuals for whom a CRP (mg/Liter) measurement was available (RSIII-1: 3,462). We considered all individuals with a clinically elevated CRP level (CRP>10 mg/Liter) as having a potential infection and excluded them from the analysis.

All analyses were performed using SPSS software (Version 21.0). Statistical significance of associations was accepted at a *P*-value < 0.05.

### Data availability

Data can be obtained upon request. Requests should be directed towards the management team of the Rotterdam Study (secretariat.epi@erasmusmc.nl), which has a protocol for approving data requests. Because of restrictions based on privacy regulations and informed consent of the participants, data cannot be made freely available in a public repository.

The Rotterdam Study has been approved by the Medical Ethics Committee of the Erasmus MC and by the Ministry of Health, Welfare and Sport of the Netherlands, implementing the “Wet Bevolkingsonderzoek: ERGO (Population Studies Act: Rotterdam Study)”. All participants provided written informed consent to participate in the study and to obtain information from their treating physicians.

## Results

### Main analysis

In total 8,711 participants were included in the analyses for the three inflammatory measures (see Supplementary Figure 1). The cohort characteristics are presented in Table 1.

**Table 1:** Cohort characteristics.

Characteristic		Study Cohort	
		N	%
Total		8,711	100
Sex	Male	3,733	42.9
	Female	4,978	57.1
Age (years)	Mean (SD)	65.9	10.5
Age category (years)	45–54	1,474	16.9
	55–64	2,780	31.9
	65–74	2,573	29.5
	75–84	1,583	18.2
	≥85	302	3.5
SES	High	1,651	19.2
	Intermediate	3,597	41.9
	Low	3,346	38.9
BMI (kg/m²)	Mean (SD)	27.1	4.1
Smoking	Current	1,734	20.2
	Former	4,288	49.9
	Never	2,570	29.9
Diabetes Status		952	10.9

SD; standard deviation, SES; socio-economic status, BMI; Body Mass Index.

Unknown: SES (117), smoking (119) and BMI (167).

Sex, SES status and BMI at baseline. Age, smoking status and DM status at time of blood draw.

To assess differences between distribution of the inflammatory markers amongst the various covariates we used the Students’ t-test or ANOVA (Analysis of Variance). All tests were statistically significant.

The mean NLR in the general population was, 1.76, with a 2.5% limit at 0.83 and 97.5% limit at 3.92. The mean NLR was statistically significantly higher in males (mean of 1.88) than in females (mean of 1.68), *P*-value < 0.001 (see Supplementary Figure 2). The mean NLR was generally higher in the higher age categories, with the highest age category >85 years of age having a mean NLR of 2.13 versus those in the youngest age category of 45–54 years of age of 1.63 (*P*-value < 0.001, Table 2). The shape of distribution of the NLR also changed with age, being almost normal for the younger age categories whilst becoming more asymmetrical with age (see Supplementary Figure 3). The Skewness statistic and standard error (SE) are: 1.4 (SE: 0.06), 2.2 (SE: 0.05), 2.6 (SE: 0.05), 2.0 (SE: 0.06) and 3.2 (SE: 0.14) for the age categories: 45–54 years, 55–64 years, 65–74 years, 75–84 years and ≥85 years, respectively.

**Table 2:** Reference values for the inflammatory markers.

			NLR	PLR	SII
General Population		mean	1.76	120	459
		2.5% limit	0.83	61	189
		97.5% limit	3.92	239	1168
Sex	Male	mean	1.88	112	453
		2.5% limit	0.88	57	185
		97.5% limit	4.14	230	1168
	Female	mean	1.68	126	463
		2.5% limit	0.80	65	194
		97.5% limit	3.80	246	1169
Age category (years)	45–54	mean	1.63	118	456
		2.5% limit	0.80	62	189
		97.5% limit	3.44	211	1063
	55–64	mean	1.61	116	436
		2.5% limit	0.79	60	186
		97.5% limit	3.53	226	1109
	65–74	mean	1.82	119	455
		2.5% limit	0.86	60	186
		97.5% limit	3.92	239	1131
	75–84	mean	2.02	127	500
		2.5% limit	0.96	61	196
		97.5% limit	4.53	268	1373
	≥85	mean	2.13	131	522
		2.5% limit	0.89	63	205
		97.5% limit	5.86	282	1798

NLR = neutrophil-to-lymphocyte ratio = absolute peripheral granulocyte count (×10^9^/L)/absolute lymphocyte count (×10^9^/L).

PLR = platelet-to-lymphocyte ratio = absolute peripheral platelets count (×10^9^/L)/absolute peripheral lymphocyte count (×10^9^/L).

SII = systemic immune-inflammation index = absolute peripheral granulocyte count (×10^9^/L)/absolute lymphocyte count (×10^9^/L). *absolute peripheral platelets count (×10^9^/L).

Similar to the NLR, both the PLR and SII were higher in the higher age categories (*P*-value < 0.001 for both). However the PLR and SII were higher in women than in men (*P*-value < 0.001 and 0.027, respectively) (see Table 2, Supplementary Figures 2 and 3). These results were consistent within the three sub-cohorts separately (data not shown).

To evaluate whether inflammatory markers indeed increase over time, we assessed the change of the inflammatory markers in 5,842 participants with two measurements. At the second blood draw the mean NLR was 1.90 and the mean SII was 465, both significantly higher (Paired Samples t-test: *P*-value < 0.001 for both). The mean PLR at the second blood draw was 119 and significantly lower compared to the first blood draw. The median within-person change was for the NLR: 0.10 (IQR: −0.21–0.44), for the PLR: −3 (−20–14) and for the SII: 19 (−72–126).

### Sensitivity analyses

To see whether the distribution was influenced by any current infection, we investigated the effect of excluding individuals with an elevated CRP level. CRP measurements were only performed for 3,462 individuals in RS-III-1, of whom in 133 individuals (3.8%) the CRP level was >10 mg/L and 3,322 (96.0%) individuals had a normal CRP level. Individuals with an elevated CRP level had a significantly higher mean NLR (2.24), PLR (129) and SII (691) compared to those with a normal CRP level; mean NLR (1.61), PLR (117) and SII (444) (Student’s t-test: *P*-value for all <0.001). However, removing individuals with an elevated CRP from the population did not affect the mean of the overall population for any of the inflammatory indices. It also only slightly affected the 97.5% limit. When individuals with a clinically elevated CRP were excluded from the population; the 97.5% limit changed from 3.60 to 3.50 (for the NLR), from 225 to 221 (for the PLR) and from 1112 to 1061 (for the SII), respectively. Individuals with an elevated CRP at the first measurement showed a decrease in the NLR levels (median −15.5%), whereas for individuals with a normal CRP, the median NLR increased with 6.3%.

## Discussion

In the past few years, novel inflammatory markers for prognosis in patients with cancer and cardiovascular disease have been described in the literature. The NLR, PLR and the SII are all composites of blood cell counts, which are standard, low-cost measurements that are already incorporated into daily clinical practice and can be calculated easily from these widely available current measures.

However, the reference limits of these white blood –cell based inflammatory markers in the general population are unknown. Therefore the cut-off values, used for risk assessment, were generally estimated in a clinical sample population consisting of patients with solid tumors. This has resulted in a wide and inconsistent range of cut-off points presented throughout the present literature. To properly evaluate the clinical significance of these new inflammatory markers we need to be able to interpret them in the context of the normal ranges. Knowledge of their distribution and reference values within the general population is therefore essential. This paper provides those reference values, obtained from a large population-based cohort aged 45 years and older.

All inflammatory markers had a skewed (right) distribution. Even when outliers with a clinically elevated CRP were excluded from the population, the distribution in the general population remained asymmetrical. The distributions also did not change when stratified for sex.

However, the distribution of the SII, NLR and PLR was different between age categories (see Supplementary Figure 3). This is especially apparent for the distribution of the NLR. The skewed distribution of inflammatory markers in the overall population can largely be attributed to the distribution amongst the higher age categories, whereas the distribution of the NLR amongst the lower age categories is almost normal. We showed that all inflammatory markers increased with age. This resembles the distribution of CRP and the ESR over different age categories^20,21^. Possibly the distribution skews with age, however it is also possible, and perhaps more likely, that its non-symmetry can be attributed to diseases that become more prevalent with age, such as diabetes, cardiovascular disease and cancer. Future research should elucidate the relationship between these inflammatory markers and morbidity in the general population.

Strengths of this study are its prospective nature, its size, and the fact that it is population based. Therefore, we obtained a good estimate of the true normal range of the inflammatory markers within the general population aged 45 years and older and additionally provided insight into the variation of these inflammatory markers. We showed that they increase with age (consistent for all three sub-cohorts) and that the reference values are different for men and women, which is consistent with current literature on CRP and ESR^20,21^. Furthermore, for the NLR and SII we showed that they increase over time.

However, there are some limitations of this study that deserve mentioning. To be able to calculate the inflammatory markers, we needed a differential white blood count. For the absolute neutrophil count we had to take the total granulocyte count as a proxy. However, any misclassification of granulocytes would probably be non-differential and therefore would not have introduced any bias into the results. Another potential limitation is that this measurement was only part of the protocol from the fourth study centre visit of the first cohort onwards, meaning that we have no information on the one-third of the population that had died before that time point. Some participants refused to give blood, meaning that in total about 40% of the original population had to be excluded from this analysis. However, we do not believe that the exclusion of this part of the study population has introduced any bias into this study, as this reflects what happens in the general population.

Although the CRP measurements are available for only a part of the population, a sufficient number remains to draw conclusions on the effect of an elevated CRP level on the inflammatory markers.

Lastly the population we examined consisted predominantly of Caucasians (98%) and raises the question whether these results are generalizable towards other ethnic groups. It is known that there are hematologic differences between, for instance, Caucasians and African-Americans^22–24^. Although our results could be used as a bench-mark, we would suggest similar studies amongst different ethnicities to further confirm these new reference values.

In conclusion, this paper provides reference values for three novel prognostic systemic inflammatory markers; the neutrophil-to-lymphocyte ratio, the platelet-to-lymphocyte ratio and the systemic immune-inflammation. This is essential to further evaluate the potential value for clinical practice of these new inflammatory markers.

## Electronic supplementary material

Supplementary Figures
