# Supplementary material for: Reference values for white blood-cell-based inflammatory markers in the Rotterdam Study: a population-based prospective cohort study
Source: Sci Rep. 2018 Jul 12;8:10566. doi: 10.1038/s41598-018-28646-w (PMC6043609; doi:10.1038/s41598-018-28646-w)
Supplement: Supplementary file 1 — Supplementary Figures [file 41598_2018_28646_MOESM1_ESM.doc]

**Supplementary Materials**

**Title:** Reference values for white blood-cell-based inflammatory markers in the Rotterdam Study: a population-based prospective cohort study.

**Authors:** Jesse Fest^1,2^, Rikje Ruiter^2^, Mohammad Arfan Ikram^2^, Trudy Voortman ^2^, Casper H.J. van Eijck^1^, Bruno H. Stricker^2^

**Affiliations:**

1. Department of Surgery, Erasmus University Medical Center, Rotterdam, the Netherlands
2. Department of Epidemiology, Erasmus University Medical Center, Rotterdam, the Netherlands

**Correspondence:** prof. dr. B.H. Stricker, Department of Epidemiology, PO Box 2040, 3000 CA Rotterdam, the Netherlands, [b.stricker@erasmusmc.nl](mailto:b.stricker@erasmusmc.nl), 0031-10-7044294.

**Figure Legend**

Supplementary Figure 1. Diagram of examination cycles of the Rotterdam Study.^15^

Supplementary Figure 2. Distributions of the inflammatory markers stratified for gender.

Panel 2. A. NLR

Panel 2. B. PLR

Panel 2. C. SII

Supplementary Figure 3. Distributions of the inflammatory markers across age categories.

Panel 3. A. NLR

Panel 3. B. PLR

Panel 3. C. SII

**Supplementary Figure 1. Diagram of examination cycles of the Rotterdam Study.** ^15^


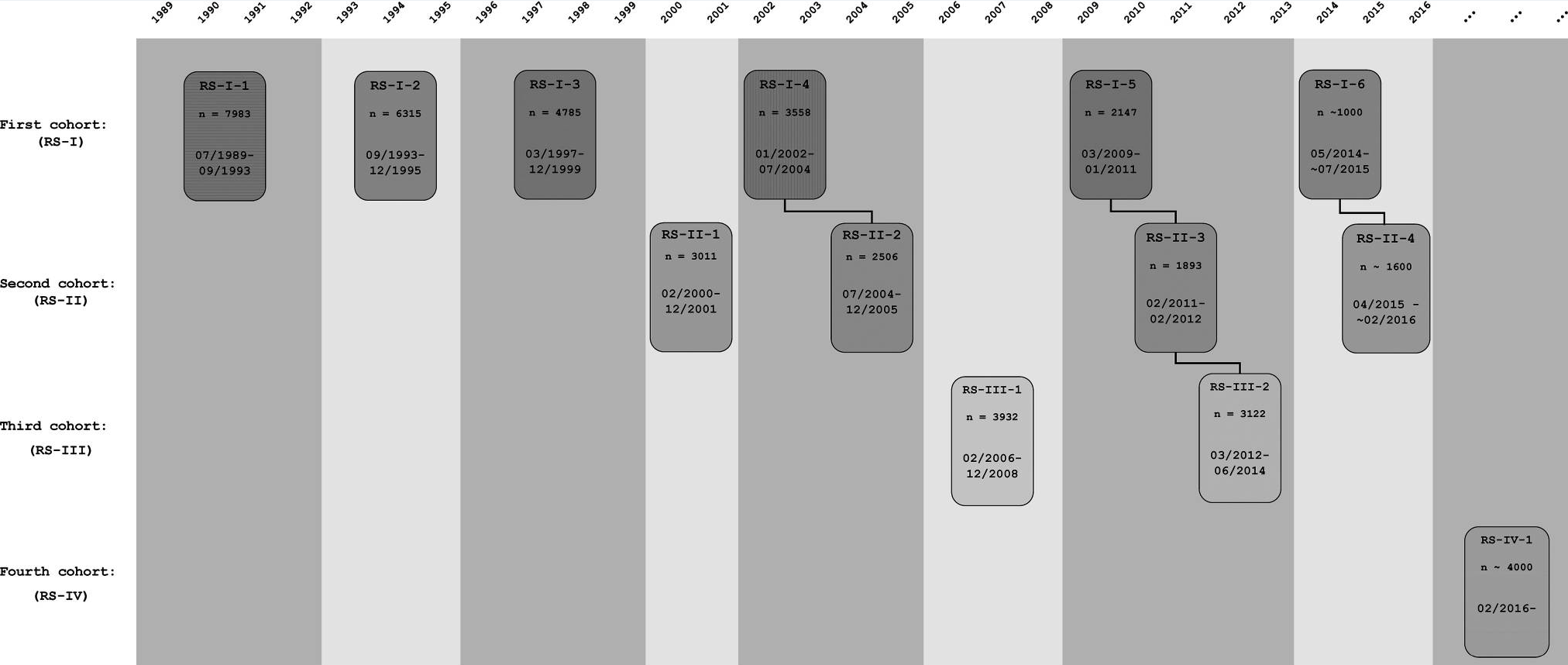


This figure was taken from Hofman et al. *Eur J Epidemiol* **30**, 661-708 (2015). Diagram of examination cycles of the Rotterdam Study (RS). RS-I-1 refers to the baseline examination of the original cohort (pilot phase 07/1989–12/1989; cohort recruitment 01/1990–09/1993). RS-I-2, RS-I-3, RS-I-4, RS-I-5, and RS-I-6 refer to re-examinations of the original cohort members. RS-II-1 refers to the extension of the cohort with persons in the study district that became 55 years since the start of the study or those of 55 years or over that migrated into the study district. RS-II-2, RS-II-3, and RS-II-4 refer to re-examinations of the extension cohort. RS-III-1 refers to the baseline examination of all persons aged 45 years and over living in the study district that had not been examined already (i.e., mainly comprising those aged 45–60 years). RS-III-2 refers to the first re-examination of this third cohort. Examination RS-I-4 and RS-II-2 were conducted as one project and feature an identical research program. Similarly, examinations RS-I-5, RS-II-3, and RS-III-2 share the same program items. Also, examinations RS-I-6 and RS-II-4 are conducted as one project. RS-IV-1 refers to the baseline visit of a new cohort, to be established in February 2016.

**Supplementary Figure 2. Distributions of the inflammatory markers stratified for gender.**

1. **NLR**


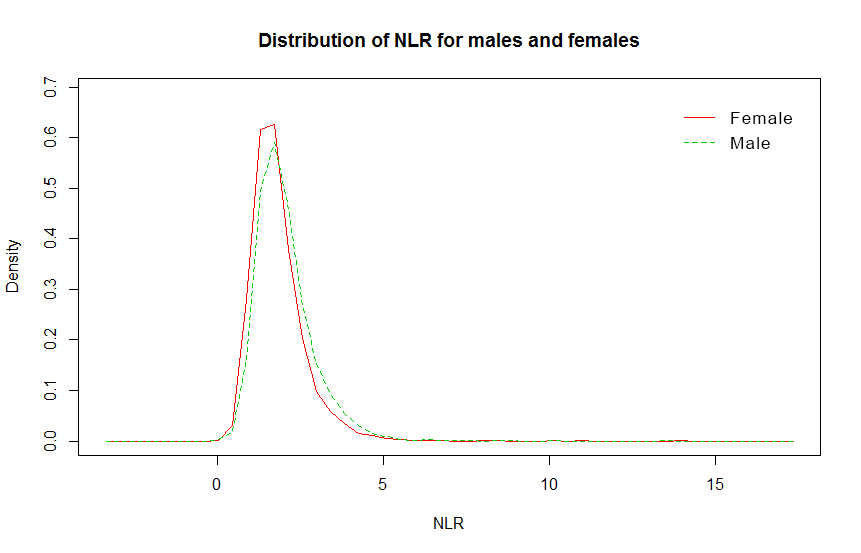


1. **PLR**


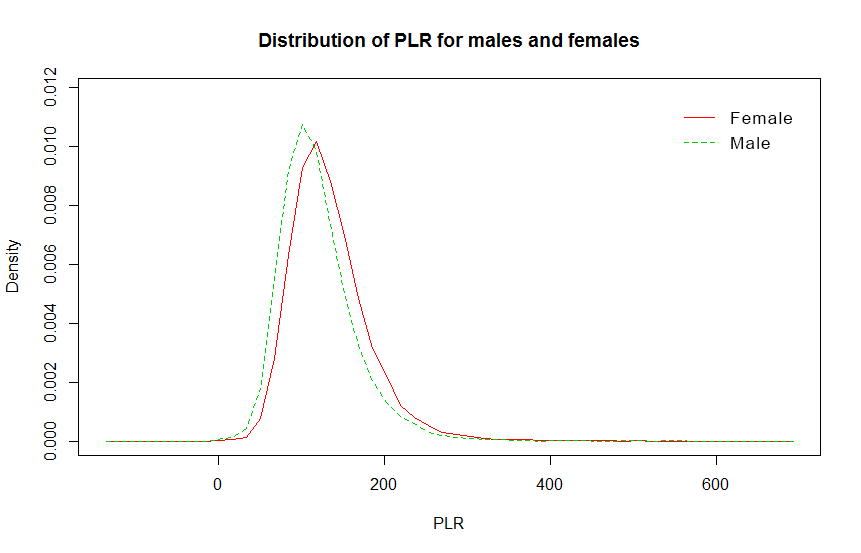


1. **SII**

**
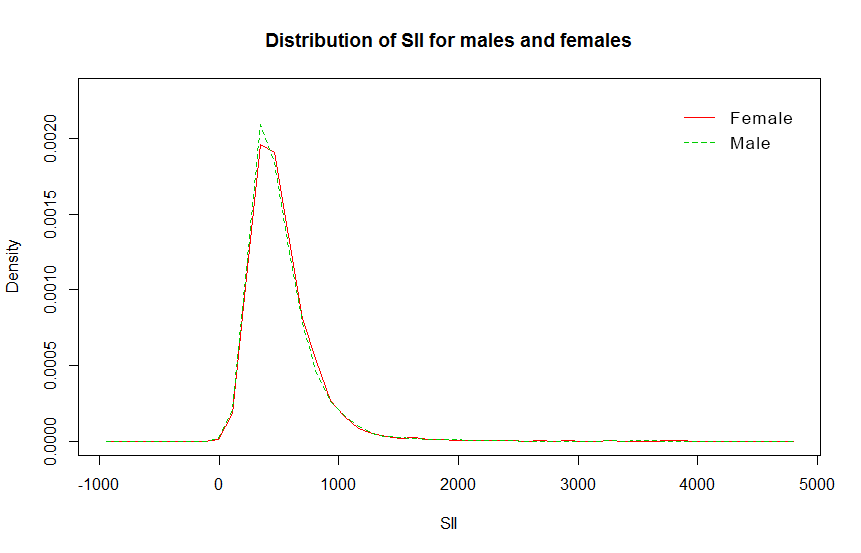
**

**Supplementary Figure 3. Distributions of the inflammatory markers across age categories.**

1. **NLR**

**
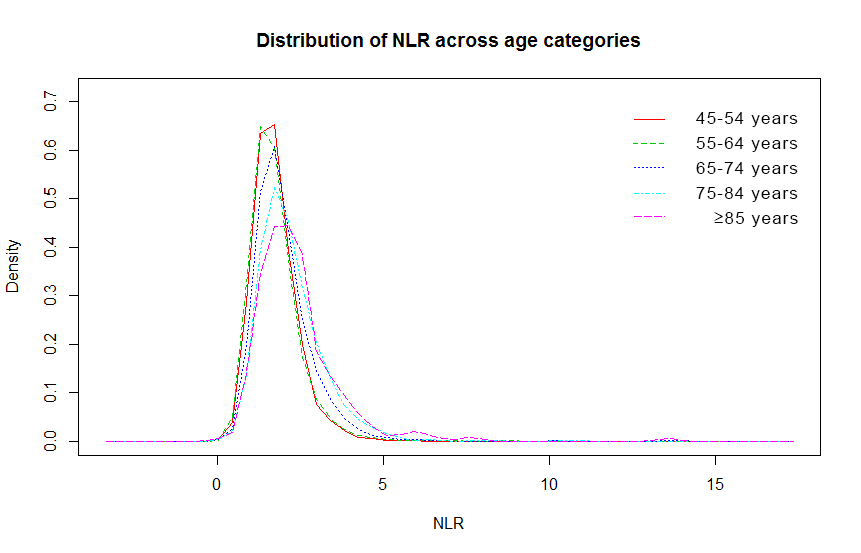
**

1. **PLR**

**
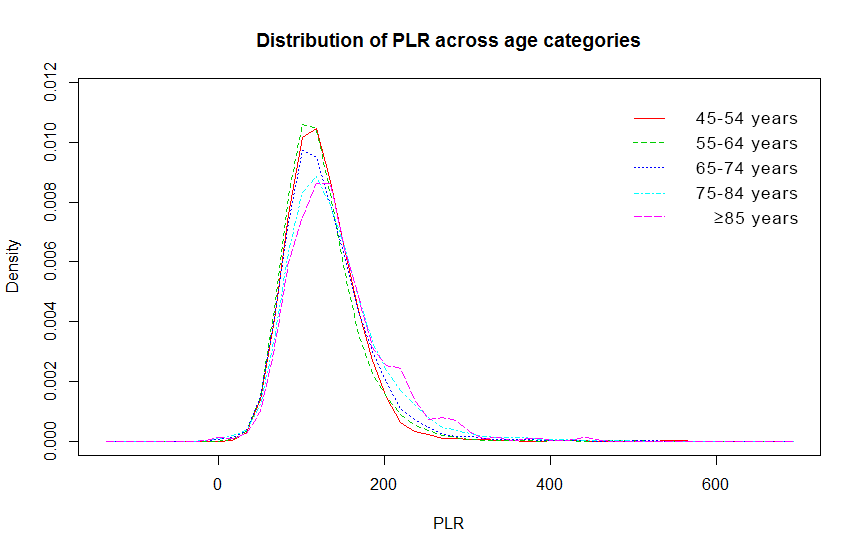
**

1. **SII**

**
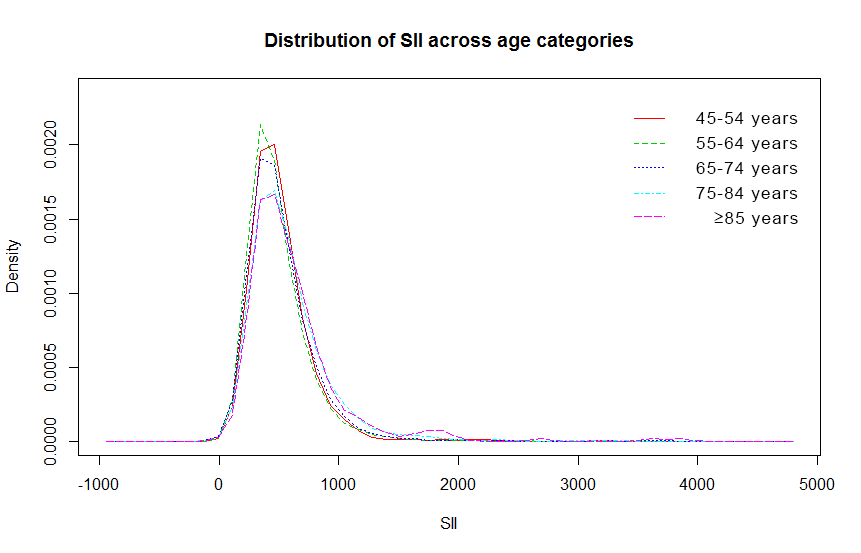
**
